# VGF expression by T lymphocytes in patients with Alzheimer's disease

**DOI:** 10.18632/oncotarget.3569

**Published:** 2015-03-14

**Authors:** Stefan Busse, Johann Steiner, Sarah Glorius, Henrik Dobrowolny, Sabrina Greiner-Bohl, Christian Mawrin, Ursula Bommhardt, Roland Hartig, Bernhard Bogerts, Mandy Busse

**Affiliations:** ^1^ Department of Psychiatry, University of Magdeburg, Magdeburg, Germany; ^2^ Center for Behavioral Brain Sciences, Magdeburg, Germany; ^3^ Department of Neuropathology, University of Magdeburg, Magdeburg, Germany; ^4^ Institute of Molecular and Clinical Immunology, University of Magdeburg, Magdeburg, Germany; ^5^ Department of Pediatric Pulmonology, Allergology & Neonatology, Medical University of Hannover, Hannover, Germany

**Keywords:** Alzheimer's disease, T cells, VGF expression, rivastigmine, rapamycin

## Abstract

Secretion of VGF is increased in cerebrospinal fluid and blood in neurodegenerative disorders like Alzheimer's disease (AD) and VGF is a potential biomarker for these disorders. We have shown that VGF is expressed in peripheral T cells and is correlated with T cell survival and cytokine secretion. The frequency of VGF+CD3+ T cells increases with normal aging. We found an increased number of VGF-expressing T cells in patients with AD compared to aged healthy controls, which was associated with enhanced HbA1c levels in blood. Upon treatment with rivastigmine, T cell proliferation and VGF expression in AD patients decreased to the level found in controls. Moreover, rapamycin treatment *in vitro* reduced the number of VGF+CD3+ cells in AD patients to control levels.

## INTRODUCTION

Epidemiological studies indicate that the population of people aged more than 65 years is constantly growing [1]. Therefore, the study of age-related diseases such as neurodegenerative or cardiovascular disorders is becoming more important. The number of patients suffering from Alzheimer's disease (AD) is expected to triple by the year 2050 [2]. AD is a progressive disorder characterized by a loss of memory and cognitive functions with behavioural alterations. The key neuropathological hallmarks of AD are extracellular amyloid plaques in the brain and intracellular neurofibrillary tangles, accompanied by the loss of neurons, white matter and synapses [3]. The plaques are often surrounded by activated microglia and inflammation may also be important in the pathogenesis of AD since increased concentrations of several inflammatory mediators, including tumor necrosis factor-alpha (TNF-α), interleukin (IL)-6, and IL-1ß have also been detected in blood [4].

In this context, the growth factor VGF may provide a link between central nervous system pathology and systemic immune and energy metabolism changes in AD. VGF is synthesized by neurons in the brain where it promotes growth and survival of neurons [5], and is involved in neurogenesis, synaptogenesis and energy homeostasis [6]. VGF is synthesized initially as a larger precursor, which undergoes proteolytic processing to produce smaller bioactive peptides that are secreted into the cerebrospinal fluid (CSF) and blood. Expression of VGF is induced by other growth factors, such as nerve growth factor (NGF), brain-derived neurotrophic factor (BDNF), neurotrophin 3, fibroblast growth factors and insulin. Since previous studies have shown that altered secretion of these factors occurs in neurological and psychiatric disorders, VGF peptides may also be biomarkers for AD, frontotemporal dementia or schizophrenia [7].

Similar to other brain-associated factors, VGF has also been detected in peripheral human leukocytes [8]. Recently, we investigated VGF expression in T cells of mentally healthy persons aged between 22 and 88 years and detected an age-dependent increase in the number of VGF+CD3+ T cells that correlated with glycated hemoglobin (HbA1c) and body mass index (BMI) [9]. T cells are known to be involved in healthy brain functions such as spatial learning, memory [10] and adult neurogenesis [11]. In addition, they have been implicated in neurodegenerative disorders like AD [12, 13] by regulating and maintaining inflammatory responses in the brain and periphery. Since currently used medications can only decelerate neurodegeneration for a certain time, new therapeutic treatment options for these disorders are needed. For example, studies have shown that the mTOR inhibitor rapamycin reduces amyloid-beta levels, abolishes cognitive deficits in mouse models of Alzheimer's disease [14] and suppresses brain aging in rats [15].

T cells are known to produce acetylcholine (ACh) [16] and may therefore be affected by ACh inhibitors that are commonly used to treat AD patients. Therefore, we analyzed T cell VGF expression in AD patients and matched healthy controls and tested for effects of treatment with the Ach inhibitor rivastigmine on the number of VGF+CD3+ T cells.

## RESULTS

### Expression of VGF and Hb1Ac levels are enhanced in AD patients

**Figure 1:**
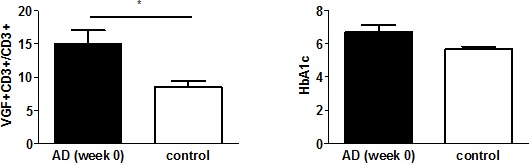
VGF+CD3+ T cells and HbA1C level are increased in AD patients The number of VGF-expressing T cells (left) and levels of HbA1c (right) were determined in the peripheral blood of 24 AD patients (week 0) and 14 neuropsychiatric healthy age-matched controls.

We determined the number of VGF+CD3+ T cells in parallel to HbA1c in AD patients, given the previous association found for these two factors in normal aging [9]. On average 8.45% of CD3^+^ T cells expressed VGF in healthy controls. However, the frequency of VGF+CD3+ T cells was significantly higher at 15.05% (p=0.032) in AD patients at the time of diagnosis (Fig. 1). The HbA1c level was determined during routine blood analysis as an 8–12 week integrated average blood glucose measurement. At the time of diagnosis, AD patients showed a trend towards higher HbA1c (6.7%) compared to controls (5.7%; p=0.060; Fig. 1).

### Expression of VGF decreases during treatment of AD patients with rivastigmine

**Figure 2:**
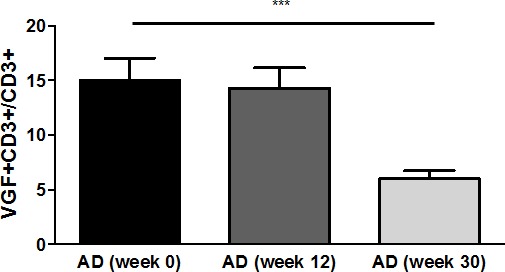
Frequency of VGF+CD3+ T cells decreases upon rivastigmine treatment Upon diagnosis of AD (week 0), treatment with rivastigmine patches was initiated. VGF expression was determined in peripheral T cells after 12 weeks and 30 weeks of constant medication.

The number of VGF+CD3+ T cells was also determined after treatment of AD patients with rivastigmine patches for 12 and 30 weeks. This showed that the VGF+CD3+ T cell percentage decreased from 15.05% at week 0 (before treatment initiation) to 14.31% at week 12 and to 6.0% at week 30 (p=0.001; Fig. 2).

### Proliferation of T cells is influenced by rivastigmine treatment

**Figure 3:**
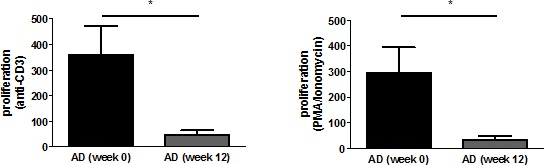
T cell proliferation decreases upon rivastigmine treatment The proliferation index was calculated by dividing the mean of anti-CD3-stimulated cultures by that of medium controls (left) and by dividing the mean of PMA/Ionomycin-stimulated cultures by that of medium controls (right).

To measure the influence of rivastigmine treatment on T cell proliferation, cells were stimulated with either anti-CD3 or a combination of phorbol 12-myristate 13-acetate (PMA) and ionomycin. The proliferation index (PI) was calculated by dividing the value of ³H-thymidine incorporation in stimulated cell cultures by that in untreated cells (medium control). The PI of anti-CD3-stimulated T cells at the time of diagnosis was 361.2 and this was reduced after the 12 week rivastigmine treatment to 48.0 (p=0.024). Stimulation with PMA/ionomycin resulted in a PI of 294.8 which was reduced to 33.4 after the treatment period (p= 0.032; Fig. 3).

### Rapamycin decreases the frequency of VGF+CD3+ cells in AD patients

**Figure 4:**
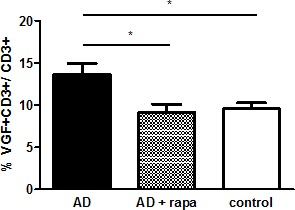
The frequency of VGF+ T cells decreases upon rapamycin treatment *in vitro*

Since Rapamycin is a suggested novel treatment for AD, we cultured PBMCs from AD patients for 24h in the presence or absence of rapamycin and determined the frequency of VGF+CD3+ T cells by flow cytometry. In cells treated without rapamycin, the percentage of VGF+CD3+ T cells was 9.56% in controls and13.65% in AD patients (p=0.0284). Treatment with rapamycin decreased the number of VGF+CD3+ T cells in AD patients to 9.1% (p=0.0197) (Fig. 4).

## DISCUSSION

In this study, we showed that VGF is expressed in a higher percentage of peripheral CD3+ T cells from AD patients compared to age-matched neuropsychiatric healthy volunteers. Upon treatment with rivastigmine, the percentage of VGF+CD3+ T cells decreased to the level found in control persons.

T lymphocytes are key components of the immune system which also exert pro-cognitive functions [17]. Brain-specific T cells could have beneficial roles in the protection against neurodegeneration by several mechanisms, such as enhancing uptake of cell debris by microglia [18, 19], release of anti-inflammatory cytokines [20, 21], increasing the capacity for buffering glutamate toxicity [22, 23], increased expression of neurotrophic factors [19, 24, 25] and enhanced neurogenesis [11, 26-28]. However, effector and regulatory functions of lymphocytes are compromised during aging [29, 30], a process called immunosenescence. Further immune manifestations accompany the progression of AD [4, 31], with negative effects on brain function and neuronal repair processes in general. This includes the deposition of extracellular amyloid plaques and intracellular neurofibrillary tangles and the associated induction of inflammatory reactions in the brain [32].

Previous studies have shown that Aβ-specific T cells are not suitable as biomarkers for AD, as the frequency of Aβ-specific T cells is significantly increased both in elderly healthy individuals and patients with AD [33]. Moreover, we have shown that an immune response against brain-specific proteins is found in patients with dementia as well as in aged people without neuropsychiatric disorders [9, 34, 35]. However, other studies have found differences in brain-specific miRNAs in blood from AD patients and healthy controls [36, 37]. Here, we found that VGF is expressed at a higher frequency in T cells from AD patients compared with those from aged-matched healthy controls. This is consistent with previous studies which found changes in the VGF level in brain or CSF from schizophrenia [38], depressive disorder [39], Parkinson's and Alzheimer's disease patients [40, 41], as compared to the respective healthy control populations. We also found previously that blocking VGF reduced the cytokine secretion and proliferation of T cells, suggesting that the enhanced frequency of VGF-expressing T cells from AD patients reflects a higher inflammatory state.

Neurotransmitters and neuropeptides can modulate the functions of immune cells such as T cells, myeloid cells or dendritic cells when released into the blood. Moreover, T cells themselves produce growth factors like NGF [42, 43], BDNF [44, 45], and insulin/insulin-like growth factors (IGF-I and IGF-II) [46]Production of these factors is increased in activated T cells, as found in inflammation [43], and may induce VGF via an autocrine mechanism. Here, we showed that treatment with the acetlycholinesterase inhibitor rivastigmine led to decreased VGF expression and T cell proliferation to the levels detected in control T cells. Treatment of cognitive impairment in AD patients [47] with acetylcholinesterase inhibitors have also been found to decrease the production of pro-inflammatory cytokines and induced the secretion of the anti-inflammatory cytokine IL-4 [48]. T cells express both muscarinic and nicotinic acetylcholine receptors and choline acetytransferase [49]. The latter synthesizes ACh which is released from T cells and acts as an immunomodulator [50]. Rivastigmine treatment stimulates ACh release which subsequently induces cholinergic receptor activation. The drug suppresses α7 nAChR-dependent the T-cell proliferation [51], as we have shown here for anti-CD3- and PMA/Ionomycin-induced T cell proliferation. However, while T cell proliferation was reduced 12 weeks after beginning of rivastigmine treatment, VGF expression was diminished only after 30 weeks. This suggests that VGF is mainly expressed by activated T cells.

Previous studies have shown that VGF peptides are involved in regulation of several functions, including energy balance homeostasis [52]. We have already described a positive correlation between HbA1C and the frequency of VGF-positive T cells [9]. Here we show that AD patients have an increased HbA1C level compared to aged-matched controls. Increased Previous studies have shown that HbA1c levels are correlated with plasma Aβ1-42 concentrations [53] and mild cognitive impairment or dementia [54]. Moreover, elevated HbA1c levels are associated with insulin resistance [56], cardiovascular diseases [54], which could also be linked to the development of neurodegenerative disorders [55]. Our data identify VGF as a biomarker for AD that involves peripheral inflammation as well as long-term glucose levels.

As with insulin and insulin-like growth factor 1 (IGF-1) signalling, the mTOR (the molecular target of rapamycin) pathway, has been implicated in aging according to the hyperfunction theory [57, 58]. Specifically, mTOR regulates cellular biomass by promoting protein translation and inhibiting autophagy. Consequently, blockade of mTOR signalling via rapamycin treatment has anti-aging and neuroprotective effects, and has been proposed as a potential therapeutic pharmacological compound to restrict neurodegenerative disorders [59] such as AD [60-62].

It is generally accepted that aging is the greatest risk factor for AD. We have already described an age-associated increase in VGF-expressing T cells in healthy volunteers [8] and here we have shown that treatment of PBMCs with both rivastigmine and rapamycin *in vitro* reduces the number of these cells in AD patients. Halloran et al. have shown that chronic inhibition of mTOR by rapamycin treatment in mice enhances learning and memory and modulates their behavior throughout their lifespan [66], an effect mediated by IL-1β and NMDA signalling [67]. Therefore, rapamycin could be an effective cognition-improving drug in AD. This may also be achieved via antioxidant and anti-inflammatory effects of rapamycin [77-80], leading to a reduction of Aβ plaques and neurofibrillary tangles, which normally contribute to the progressive cognitive deficits of AD [70-72]. The present findings suggest that peripheral VGF levels could be used as a biomarker for detection of AD and for monitoring treatment response with rivastigmine and rapamycin.

## MATERIALS AND METHODS

### Study population

The study was performed in accordance with German laws, the Declaration of Helsinki and guidelines of the local institutional review board. Written consent was obtained from all patients and healthy controls. We collected 18 ml blood from 24 AD patients and 14 matched controls without neuropsychiatric disorders (suppl. table 1). Routine blood analysis were performed, including differential blood cell count, levels of C-reactive protein, glucose, lipids, liver enzymes and thyroid hormones. None of the subjects were excluded due to changes in these routine blood parameters. Also, no person had a history of autoimmune disorders, immunomodulating treatment, cancer, chronic terminal disease, severe cardiovascular disorder, substance abuse or severe trauma.

### Treatment of AD patients

Within the first six weeks after diagnosis, AD patients received patches with 4.6 mg rivastigmine, followed by patches with 9.5 mg rivastigmineuntil the end of the study. Patches were changed each day.

### Preparation of peripheral blood monocyte cells (PBMC)

Blood was collected in BD Vacutainer lithium heparin-treated tubes (BD Biosciences; San Jose, CA, USA), and diluted 1:1 with phosphate-buffered saline (PBS). Peripheral blood mononuclear cells (PBMCs) were prepared by density gradient centrifugation on Ficoll Paque (Biochrom AG, Berlin) at 375g for 20 min at room temperature. The cells were harvested and washed twice in PBS. Cells were then suspended in Fluorescence Assisted Cell Sorting (FACS) staining buffer (PBS w/0.5% BSA) and cell numbers determined.

### Flow cytometry

Isolated PBMCs were washed in PBS and incubated with fluorescently-labeled antibodies for 20 min at 4°C in FACS buffer. Antibodies (Abs) used in this study were anti-CD3 (UCHT1) from BD Pharmingen (San Diego, CA, USA), and anti-VGF (D-20) and donkey anti-goat IgG FITC from Santa Cruz Biotechnology (Dallas, TX, USA). Data were collected on a FACS LSR-Fortessa (BD Biosciences, Mountain View, CA, USA) and analyzed using FlowJo software (Treestar Inc., Ashland, OR, USA). Data were analyzed using biexponential transformation for complete data visualization.

### Measurement of cell proliferation

CD3+ T cells were isolated using the Pan T cell kit (Miltenyi Biotech, Bergisch Gladbach, Germany) and AutoMacs separation. CD3+ T cells were resuspended in RPMI 1640 medium supplemented with 10% FCS and 1% antibiotics and plated in triplicate at a density of 1×10^6^ cells/ml in 96-well plates. T cells were stimulated with PMA/Ionomycin (1mg/ml) and anti-CD3 (0.5μl/100μl; eBioscience, San Diego, CA, USA) for 3 days. ³H-Thymidine (0.2μCi/well) was added to the cultures for the last 17 hours Then cells were harvested and ³H-Thymidine incorporation was measured as an index of lymphocyte proliferation in a betaplate liqid scintillation counter (Wallac). Proliferation of T cells was calculated by division of the mean value of each triplicate of anti-CD3- or PMA/Ionomycin-stimulated cell cultures by that of control medium cultures.

### Treatment of PBMC with rapamycin *in vitro*

Isolated PBMCs from AD patients and controls were cultured for 24h in complete RPMI medium with or without addition of rapamycin (200 ng/ml). The cells were harvested and expression of VGF on CD3+ T cells was measured using flow cytometry.

### Statistical analysis

Diagnostic group differences were calculated using Student's t test or ANOVA. Significance was defined as p< 0.05, while a probability level of 0.05≤ p< 0.10 was considered as a statistical trend.

## SUPPLEMENTARY MATERIAL TABLE


