# Supplementary material for: VGF expression by T lymphocytes in patients with Alzheimer's disease
Source: Oncotarget. 2015 Mar 14;6(17):14843–51. doi: 10.18632/oncotarget.3569 (PMC4558119; doi:10.18632/oncotarget.3569)
Supplement: Supplementary file 1 [file oncotarget-06-14843-s001.pdf]

VGF expression by T lymphocytes in patients with Alzheimer's disease

Supplementary Material

suppl. table: Demographic data of the study cohort

**characteristics**		**mean**	
		**AD patients**	**healthy volunteers**
**total**		24	14
**age (years)**		80.75	73.71
**gender**	**female**	19	9
	**male**	5	5
